# Mycobacterial Lipoprotein Z Triggers Efficient Innate and Adaptive Immunity for Protection Against *Mycobacterium tuberculosis* Infection

**DOI:** 10.3389/fimmu.2018.03190

**Published:** 2019-01-16

**Authors:** Yingying Chen, Jia-ni Xiao, Yong Li, Yang-jiong Xiao, Yan-qing Xiong, Ying Liu, Shu-jun Wang, Ping Ji, Guo-ping Zhao, Hao Shen, Shui-hua Lu, Xiao-yong Fan, Ying Wang

**Affiliations:** ^1^Department of Microbiology and Immunology, Shanghai Jiao Tong University School of Medicine, Shanghai Institute of Immunology, Shanghai, China; ^2^Key Laboratory of Medical Molecular Virology of MOE/MOH, Shanghai Public Health Clinical Center, Fudan University, Shanghai, China; ^3^Shanghai-MOST Key Laboratory of Health and Disease Genomics, Chinese National Human Genome Center at Shanghai, Shanghai, China; ^4^Department of Microbiology, Perelman School of Medicine, University of Pennsylvania, Philadelphia, PA, United States; ^5^TB Center, Shanghai Emerging and Re-emerging Infectious Disease Institute, Fudan University, Shanghai, China

**Keywords:** lipoprotein Z, tuberculosis, innate immunity, T cell immunity, immune protection

## Abstract

Mycobacterial lipoproteins are considered to be involved in both virulence and immunoregulatory processes during *Mycobacterium tuberculosis* (*M.tb*) infection. In our previous investigations on the immunoreactivity of more than 30 *M.tb* proteins in active TB patients, we identified mycobacterial lipoprotein Z (LppZ) as one of the most immune dominant antigens. How LppZ triggers immune responses is still unclear. In this study, we analyzed LppZ-mediated innate and adaptive immunity using a murine air pouch model and an *M.tb* infection model, respectively. We found that LppZ could not only recruit inflammatory cells but also induce the production of proinflammatory cytokines inside the pouches. LppZ could also induce strong Th1 responses following immunization and confer protection against challenge with *M.tb* virulent strain H37Rv at a similar level to BCG vaccination but with less pathological damage in the lungs. Furthermore, we revealed the presence of LppZ-specific functional CD4^+^ T cells in the lungs of the challenged mice that were capable of secreting double or triple cytokines, including IFN-γ, IL-2, and TNF-α. Our study thus demonstrates that LppZ is of strong immunogenicity during *M.tb* infection in both humans and mice and has the ability to trigger effective innate and cellular immunity. Considering the limitations of candidate antigens in the pipeline of TB vaccine development, LppZ-mediated immune protection against *M.tb* challenge in the mouse model implies its potential application in vaccine development.

## Introduction

Tuberculosis (TB), caused by *Mycobacterium tuberculosis* (*M.tb*) infection, remains one of the leading infectious diseases worldwide. The high prevalence of latent tuberculosis infection (LTBI), resulting from incomplete clearance after initial *M.tb* infection, contributes to the high incidence of active TB in epidemiological regions (1). Therefore, in addition to the requirement for rapid diagnosis and treatment, vaccine development to prevent *M.tb* infection and the reactivation of LTBI are urgently needed for TB control. Bacille Calmette-Guérin (BCG), the live attenuated *Mycobacterium bovis* strain, is the only licensed TB vaccine at present (2). Although it provides protection against miliary and meningeal TB in childhood, it exhibits less efficacy in preventing pulmonary TB in both teenagers and adults (3). Several TB vaccine candidates have entered the pipeline for clinical trials (4). However, the antigens being used for vaccine development are still very limited in number and effect. Ag85 family members are the most investigated, including Ag85A, Ag85B, and Ag85C (5, 6). Other mycobacterial antigens, such as Rv0733 (7), Rv3615c (8), HspX (9), and Mtb8.4 (10) are also under investigation for their potential as vaccine candidate antigens with no obvious success. A recent successful phase 2b trial on M72/AS01E (Mtb32A and Mtb39A) highlights the incentive of exploring new antigens in TB vaccine design (11). Therefore, it is still of paramount importance to screen and identify novel candidate antigens that have high immunogenicity and appropriate immune-regulatory activity for the purpose of TB vaccine design.

In a previous study, we have investigated more than 30 *M.tb* derived proteins for cellular immune responses in patients with active TB (7). Amongst them, lipoprotein Z (LppZ) was identified as one of the most immune dominant antigens (7). LppZ, encoded by *rv3006*, belongs to lipoprotein family with around 100 members in *M.tb* (12). It was found prevalent in a range of mycobacterium species, including *M. leprae, M. smegmatis, M. marinum*, and *M. bovis*, and was detectable in both the membrane fraction and in the culture supernatant of *M.tb* (13–15). LppZ was demonstrated to have the highest antibody reactivity in TB patients when compared to Ag85A or other lipoproteins such as PstS-1 (38 kDa antigen) and PstS-2, which are also abundant among culture filtrate proteins (14, 16). Furthermore, LppZ was shown to be sero-reactive in both cavitary and non-cavitary TB patients and to have sero-diagnostic potential for TB (17). In view of its strong immune responsiveness in active TB patients, whether and how LppZ might exert immune regulation was considered worthy of further exploration.

In the present study, T cell responsiveness to LppZ was extensively analyzed in active TB patients. Subsequently, the immunogenicity and protective efficacy of LppZ were determined systematically in murine models to provide initial evidence of the potential of LppZ as a new TB vaccine candidate antigen.

## Materials and Methods

### Study Subjects

All pulmonary TB patients (*n* = 86) and healthy volunteers (*n* = 48) enrolled in this study were adults vaccinated with the BCG Shanghai strain (Shanghai Institute of Biological Products Co. Ltd., Shanghai, China) at birth. TB patients were in-patients of Shanghai Public Health Clinical Center (SHAPHC, Shanghai, China) and signed voluntary informed consents in accordance with the Declaration of Helsinki. The healthy volunteers were defined as having no medical history of *M.tb* infection, a normal physical examination (including blood test, serum chemistry, chest X-rays) with no disease symptoms and no evidence of latent *M.tb* infection (negative in T-SPOT.TB assay). The study was approved by the Ethical Committee of Shanghai Jiao Tong University School of Medicine (SJTUSM).

### Mice and Ethics Statement

C57BL/6 female mice (6–8 weeks old) were purchased from Shanghai Laboratory Animal Center (Shanghai, China) and maintained under specific-pathogen-free (SPF) conditions in the animal facility of SJTUSM. The protocols of animal experiments were approved by the Animal Ethics Committee of SJTUSM, and were performed under the Guide for the Care and Use of Laboratory Animals.

### Expression and Purification of Recombinant *M.tb* Antigens

The recombinant plasmids were obtained from NIH BEI Research Resources Repository (NIAID, NIH), including pMRLB.7 containing *rv3875* (Protein ESAT6) (NR-36431), pMRLB.41 containing *rv3804c* (Protein Ag85A) (NR-13292), pMRLB.46 containing *rv3874* (Protein CFP-10) (NR-13297) and pMRLB.54 containing *rv3006* (Protein LppZ) (NR-13304) from *M.tb*. An *ESAT6* and *CFP-10* fusion gene (*E6C10*) was prepared through co-cloning into expressing vector pET-28α. Recombinant expressing plasmids were transformed to *Escherichia coli* BL21 (DE3) strain, and the proteins were induced with isopropyl-β-D-thiogalactoside (IPTG) (Beyotime, Jiangsu, China) for 4 h at 30°C. Bacteria were lysed and the proteins were purified by affinity chromatography with Ni-NTA His-Bind Resin (Merck Millipore, Darmstadt, Germany) as previously described (18). The purity of the proteins was monitored by 12% SDS-PAGE and further confirmed by Western blotting using anti-LppZ or anti-Ag85A mouse polyclonal antibodies and goat anti-mouse IgG antibody (Cwbio, Beijing, China). The protein bands on PVDF membrane (Bio-Rad, Hercules, CA) were visualized by adding ECL solution (Millipore, Boston, MA). The membranes were scanned on a Molecular Imager ChemiDoc XRS+ machine (Bio-Rad). Endotoxins were removed by using Triton X-114 (Sigma Aldrich, St. Louis, MO) two-phase separation as previously described (18). The remaining endotoxin in purified proteins was detected by using Tachyleus Amebocyte Lysate Kits (Gulangyu, Xiamen, China) according to the manufacturer's protocol. To verify the absence of LPS interference in the functional assay, an *in vitro* stimulation experiment was performed on Raw264.7 cells where the cells were incubated with DMEM medium (GIBCO, Grand Island, NY), LppZ (10 μg/mL) plus Polymyxin B (PMB, 10 μg/mL, Merck), lipopolysaccharide (LPS, 500 ng/mL, Sigma Aldrich), or with LPS plus PMB. After 24 h of incubation, cell culture supernatants were collected, and TNF-α and IL-6 levels were measured by ELISA kits (eBioscience, San Diego, CA).

### Murine Air Pouch Model

Dorsal air pouches were induced in mice. In brief, 4 mL sterile-filtered air was injected subcutaneously into the back of C57BL/6 female mice. After 3 days, the pouches were re-inflated with 3 mL sterile air. Three days later 1 mL 0.5% carboxymethylcellulose (CMC) (Sigma Aldrich) mixed with 50 μg LppZ or Ag85A was injected. Injection of 1 mL 0.5% CMC served as a negative control. At 4 h and 24 h post-injection, mice were sacrificed and the pouches were lavaged with 3 mL sterile PBS. The lavage fluid was centrifuged at 486 × *g* for 8 min at 4°C. Cells and supernatants were collected separately and subjected to further analyses.

### Cellular Analysis in the Pouches

Cells collected from the pouches were labeled with FITC-anti-mouse CD11b, PE-anti-mouse F4/80 (both from Biolegend, San Diego, CA) and APC-anti-mouse Ly6G (BD Biosciences, Franklin Lakes, NJ) antibodies, and acquired with a FACS CantoII flow cytometer (BD Biosciences). Data were analyzed by FlowJo software 7.5 (FlOWJO LLC, Treestar Inc., Ashland, OR). Neutrophils were defined as CD11b^+^Ly6G^hi^F4/80^−^ and macrophages as CD11b^+^F4/80^hi^Ly6G^−^.

### Myeloperoxidase Assay

Leukocyte myeloperoxidase (MPO) activity in the exudates was detected by measuring the H_2_O_2_-dependent oxidation of 3,3′,5,5′-tetramethylbenzidine (TMB) as described previously (19). Aliquots of 30 μL supernatants were incubated with 120 μL TMB substrate (eBioscience) for 5 min at room temperature. The reaction was stopped by adding 2N H_2_SO_4_. The optical density was measured at 450 nm in a PowerWaveXS2 microplate spectrophotometer (BioTek Instruments Inc., VT). All the samples were assayed in duplicate.

### Mouse Immunization and *M.tb* H37Rv Challenge

C57BL/6 female mice (12 mice per group) were immunized subcutaneously with 10 μg LppZ or Ag85A emulsified with adjuvant mixture containing 250 μg dimethyl dioctadecylammonium bromide (DDA) and 25 μg monophosphoryl lipid A (MPL) (both from Sigma Aldrich) in 200 μL PBS three times with 2-weeks intervals as previously described (7). Mice immunized with the adjuvant mixture only were treated as a negative control, while one dose of 5 × 10^6^ BCG Shanghai strain (Shanghai) served as a positive control. Four weeks after the final immunization, mice were subjected to immunological analysis (6 mice per group) or H37Rv challenge (6 mice per group).

For H37Rv challenge, mice were exposed to 100–200 colony-forming units (CFUs) bacteria in an automatic inhalation system (Glas-Col Model #A4212 099c, Terre Haute, IN) in the ABSL-3 animal facility at SHAPHC (7). At 4 weeks post-challenge, mice were sacrificed and the lobes of the lungs were separated for enumerating bacterium CFU, hematoxylin and eosin (H&E) staining, and immunological analysis. More specifically, part of the lungs from infected mice including left lung, right middle and inferior lobes, were homogenized to calculate bacterial numbers as previously described (20). The right post-caval lobes from each mouse were used for H&E staining. The right superior lung lobes were used for immunological analysis. Bacterial loads in the spleens were determined by CFU counting.

### Isolation of Human Peripheral Blood Mononuclear Cells (PBMCs)

Heparin anticoagulant human peripheral blood (5–10 mL) was collected. PBMCs were isolated by Ficoll-hypaque density-gradient centrifugation with Lymphoprep^TM^ solution (Axis Shield, Oslo, Norway) at 800 × *g* for 20 min at room temperature. The mononuclear cell layer was collected and washed once with RPMI 1640 culture medium (GIBCO). PBMCs were resuspended in cell culture medium (RPMI 1640 culture medium containing 10% fetal bovine serum (FBS), 100 U/mL penicillin, and 100 μg/mL streptomycin) (all from GIBCO) and subjected to further immunological analysis.

### Harvest of Mouse Splenocytes and Pulmonary Immune Cells

The lungs of bacteria-challenged mice were finely minced and digested with 1 mg/mL Collagenase IV (Life Technologies, Waltham, MA) and 200 μg/mL DNase I (Fermantas, Waltham, MA). Single cell suspensions were obtained through 0.15 mm sieves. Pulmonary lymphocytes and splenocytes from immunized mice were isolated by Ficoll-hypaque density gradient centrifugation. Cells were resuspended in mouse cell culture medium (RPMI 1640 culture medium containing 10% FBS, 50 μM 2-mercaptoethanol, 1 mM L-glutamine, 100 U/mL penicillin, and 100 μg/mL streptomycin) for further immunological analysis.

### Antigen-Specific Interferon-Gamma (IFN-γ) Release Assay

Antigen-specific IFN-γ release was detected by enzyme-linked immunospot (ELISpot) assay according to the manufacturers' instructions (human IFN-γ ELISpot kit: U-CyTech, Utrecht, Netherlands; mouse IFN-γ ELISpot kit: BD Biosciences). Briefly, 96-well PVDF plates (Millipore) were coated with anti-human or anti-mouse IFN-γ coating antibody overnight at 4°C. Freshly isolated cells (2.5 × 10^5^ in 200 μL culture medium) were incubated with 10 μg/mL *M.tb* antigen (LppZ, E6C10 fusion protein, Ag85A), tuberculin PPD (Statens Serum Institut, SSI, Copenhagen, Denmark), 5 μg/mL Concanavalin A (ConA) or 2.5 μg/mL phytohemagglutinin (PHA) (both from Sigma Aldrich) for 20 h, respectively. IFN-γ secreting cells were detected by biotin-labeled detection antibody and horseradish peroxidase (HRP) conjugated streptavidin. Coloration was developed with AEC substrate solution for 30 min at room temperature in the dark. The reaction was stopped by thoroughly rinsing the PVDF membrane with demineralized water. The plates were air-dried and the spots were counted by ELISpot Reader (BioReader Model 4000; Bio-Sys GmbH, Karben, Germany). The number of antigen-specific IFN-γ producing cells was calculated as spot-forming units (SFUs) per 2.5 × 10^5^ cells.

### Detection of Cytokine Secretion in Mouse Lymphocytes

Freshly isolated splenocytes or pulmonary immune cells were resuspended at a concentration of 1 × 10^7^cells/mL. Cells were stimulated with 10 μg/mL antigens (LppZ and Ag85A) or tuberculin PPD for 20 h or treated with 50 ng/mL phorbol 12-myristate 13-acetate (PMA) and 500 ng/mL Ionomycin (both from Sigma Aldrich) for 5 h. GolgiStop solution (BD Biosciences) was added to cell culture during the last 5 h of *ex vivo* stimulation. For surface staining, cells were incubated with PE-Cy5-anti-mouse CD3 (eBioscience), Pacific Blue-anti-mouse CD4 (Biolegend), APC-Cy7-anti-mouse CD8 antibodies, biotin-Annexin V and PerCP-streptavidin (BD Biosciences) for 30 min in the dark at 4°C. After washing with PBS containing 2% FBS, cells were fixed and permeated by the Cytofix/Cytoperm reagent from Fixation/Permeabilization kit (BD Biosciences). Cells were incubated with FITC-anti-mouse IFN-γ, PE-anti-mouse IL-2 (both from BD Biosciences), and APC-anti-mouse TNF-α (Biolegend) antibodies for 45 min at 4°C. Cells were washed, resuspended in PBS and acquired with a FACS CantoII flow cytometer (BD Bioscience) in 2 h. Data were analyzed by using FlowJo software 7.5 (Treestar Inc.).

### Pathological Analysis of Lungs From H37Rv Infected Mice

The right post-caval lobe of the right lung was fixed in 4% neutral-buffered paraformaldehyde (PFA) solution for 24 h. The tissues were embedded with paraffin and sectioned with a thickness of 5 μm. H&E staining was performed to detect tissue damage, lymphocyte infiltration, and local inflammation in the lungs. Slides were analyzed in a double-blinded fashion by a pathologist using QwinPlus software of Leica microscope (Leica Model DMI3000 M; Leica, Wetzlar, Germany). The score was obtained by measuring the consolidation area of the whole field of vision (amplification 40) and calculated by the following formula: total area of consolidation/area of whole field of vision × 100%. The results were recorded as the mean of at least five fields from each group (n=6).

### Cytokine Detection by ELISA

Enzyme-linked immunosorbent assay (ELISA) was performed to detect IL-1β, IL-6, and TNF-α in the supernatants from air pouch lavages according to the manufacturer's instructions (all kits were from eBioscience).

### Statistical Analysis

All the data were shown as mean ± SEM. Statistical analyses were performed by using GraphPad Prism 5 software (Graphpad software Inc., CA, USA). Statistical differences were assessed by the unpaired *t*-test for the data with Gaussian distribution and by Mann-Whitney test for those with non-Gaussian distribution. Unless stated, *p* < 0.05 was considered statistically significant.

## Results

### LppZ Displays Strong Antigenicity in Both Onset and Retreated Active TB Patients Which Is Comparable to ESAT6 and CFP-10

**Figure 1:**
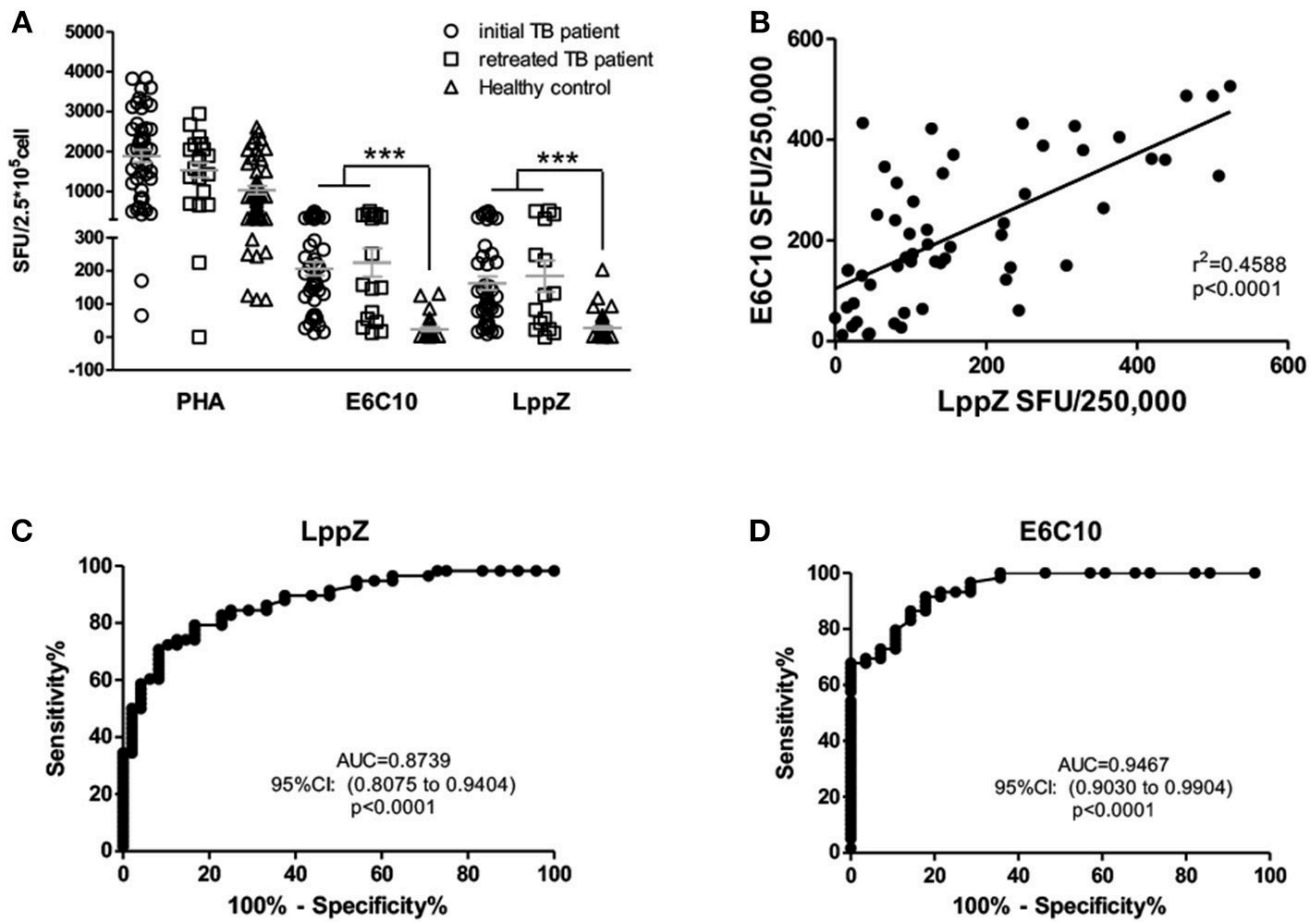
LppZ induces high cellular responsiveness in TB patients**. (A)** The numbers of IFN-γ producing cells in TB patients (including initial TB patients and retreated TB patients) and HCs were assessed by ELISpot assay after stimulation with E6C10 fusion protein or purified LppZ protein. PHA served as a positive control. **(B)** Correlation analyses between LppZ- and E6C10-specific IFN-γ producing cells. **(C,D)** ROC curve analyses of LppZ- **(C)** and E6C10-specific **(D)** IFN-γ-producing cells in distinguishing TB patients and HCs. ***p < 0.0001.

To verify the high antigenicity of LppZ in active TB patients (7, 21), LppZ was expressed prokaryotically and purified by affinity chromatography followed by the removal of endotoxin. The purity of LppZ was determined by Coomassie blue staining and validated by Western blotting using anti-6 × His tag antibody, and mouse and rabbit anti-LppZ polyclonal antibodies (Supplementary Figure 1). The numbers of LppZ-specific IFN-γ producing cells in the periphery of TB patients and healthy controls (HCs) were detected by an ELISpot assay upon stimulation with purified LppZ (22) (Supplementary Figure 2). A fusion protein of ESAT6 and CFP-10 (E6C10) was used to determine *M.tb*-specific immune status in TB patients, while PHA was used as a positive control (Figure 1A). Our results showed that both initial and retreated TB patients had significantly higher responsiveness than the HCs specific to LppZ. They had more IFN-γ producing cells, which was similar to the differential responses to E6C10 (Figure 1A). Correlation analysis revealed that TB patients responded to LppZ in a manner parallel to E6C10 (Figure 1B). Since LppZ induced strong IFN-γ production similar to E6C10 in the active TB patients, receiver operating characteristic (ROC) curve analysis was performed to evaluate the diagnostic potential of LppZ-specific IFN-γ production in TB. It was obvious that LppZ-induced cellular response was able to efficiently discriminate TB patients from HCs with the area under the curve of ROC (AUC) value of 0.8739. The sensitivity and specificity were 50% and 97.92%, respectively (Figure 1C), which was similar to the results with E6C10 (Figure 1D). LPS interference in LppZ protein stimulation was excluded (Supplementary Figure 3).These results revealed that LppZ had high antigenicity for cellular immunity in active TB patients.

### LppZ Triggers Proinflammatory Responses in a Mouse Air Pouch Model

**Figure 2:**
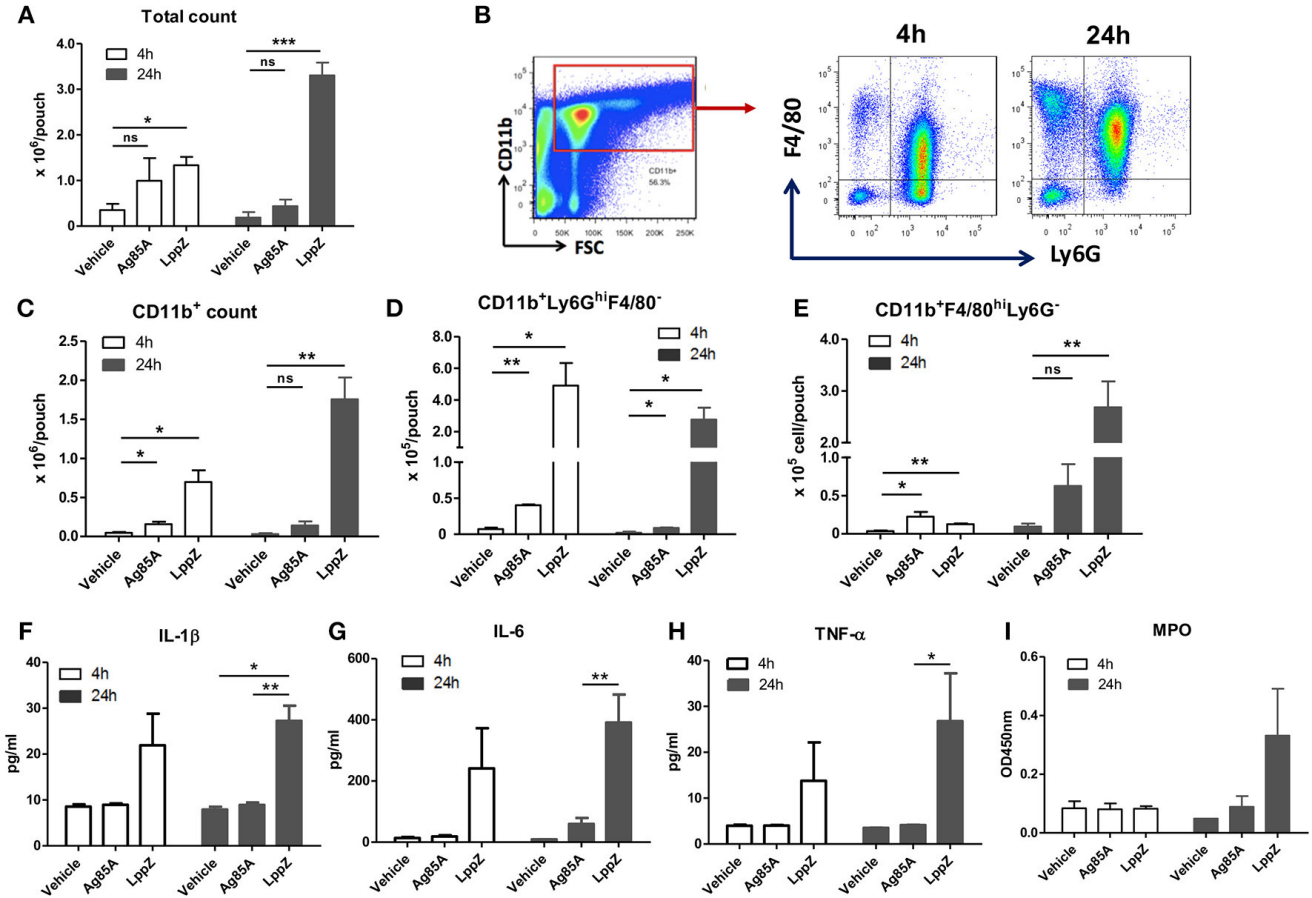
LppZ recruits innate cells and induces pro-inflammatory cytokine production in the murine air pouch model. After injecting CMC (vehicle), LppZ (LppZ) or Ag85A (Ag85A) recombinant protein in the pouches, mice were sacrificed at 4 h and 24 h. Cells in the pouches were collected. **(A)** Total cell numbers recruited in the pouches. **(B)** Representative graphs showing the gating strategy for analysis of infiltrating cell subsets. The absolute numbers of CD11b^+^ cells **(C)**, CD11b^+^Ly6G^hi^F4/80^−^ neutrophils **(D)** and CD11b^+^Ly6G^−^F4/80^+^ macrophages **(E)** were analyzed by flow cytometry. The levels of IL-1β **(F)**, IL-6 **(G)**, TNF-α **(H)**, and myeloperoxidase (MPO) **(I)** were measured in the exudates (*n* = 3–6 mice per group). *p < 0.05, **p < 0.01, ***p < 0.001.

To explore the regulation of strong cellular immune responses by LppZ, a mouse air pouch model (Supplementary Figure 4) was applied to recapitulate the innate immunity initiated at the early stage upon *M.tb* infection. Sterile-filtered air (4 mL) was injected subcutaneously to mechanically disrupt the connective tissues for the formation of the pouches. This facilitated the infiltration of immune cells. By injecting target antigens into the pouches to trigger the host innate response the characteristics of the subsequent inflammation could be further investigated (23–25). As shown in Figure 2A, injection of LppZ in 0.5% CMC drove a dramatically greater accumulation of inflammatory cells into the pouches when compared to the CMC-only group. Interestingly, injection of Ag85A in 0.5% CMC induced moderate cell infiltration inside the pouches, much less than when LppZ was used.

The types of immune cells recruited in the pouches were further defined (Figure 2B). LppZ injection led to the increased infiltration of CD11b^+^ myeloid cells when compared to other groups (Figure 2C). More precisely, at 4 h the CD11b^+^Ly6G^hi^F4/80^−^ neutrophils were much more abundant than the CD11b^+^Ly6G^−^F4/80^hi^ macrophages in both LppZ- and Ag85A-treated pouches whereas the absolute numbers of both cells were increased more in LppZ-treated pouches than in Ag85A-treated pouches (Figures 2D,E, white bar). At 24 h after either LppZ or Ag85A treatment the absolute number of the macrophages was increased whereas that of the neutrophils in the pouches decreased. Although Ag85A treatment induced macrophage accumulation in the pouches, the increase in macrophages was more dramatic in LppZ-injected than in Ag85A-injected pouches (Figures 2D,E, black bar). Few neutrophils and macrophages were detectable in vehicle-treated groups. Furthermore, when key pro-inflammatory cytokines were measured in the exudates of the pouches there were significantly higher levels of IL-1β, IL-6 and TNF-α upon LppZ treatment when compared to vehicle or Ag85A-treated groups. Interestingly, no dramatic difference in pro-inflammatory cytokines was observed between vehicle- and Ag85A-injected pouches (Figures 2F–H). Myeloperoxidase (MPO) is one of the key enzymes released by inflammatory cells during inflammation and, consistent with the dramatic recruitment of inflammatory cells and the augmented pro-inflammatory cytokine release in the pouches upon LppZ injection, the MPO level also increased significantly in LppZ-induced exudates at 24 h whereas vehicle or Ag85A treatment induced little MPO in the pouches (Figure 2I). Thus, these results strongly suggested that LppZ triggers inflammatory responses largely through the recruitment of inflammatory cells and the induction of pro-inflammatory cytokine production.

### LppZ Induces Strong Th1 Responses With Enhanced T Cell Multi-Functionality in Immunized Mice

**Figure 3:**
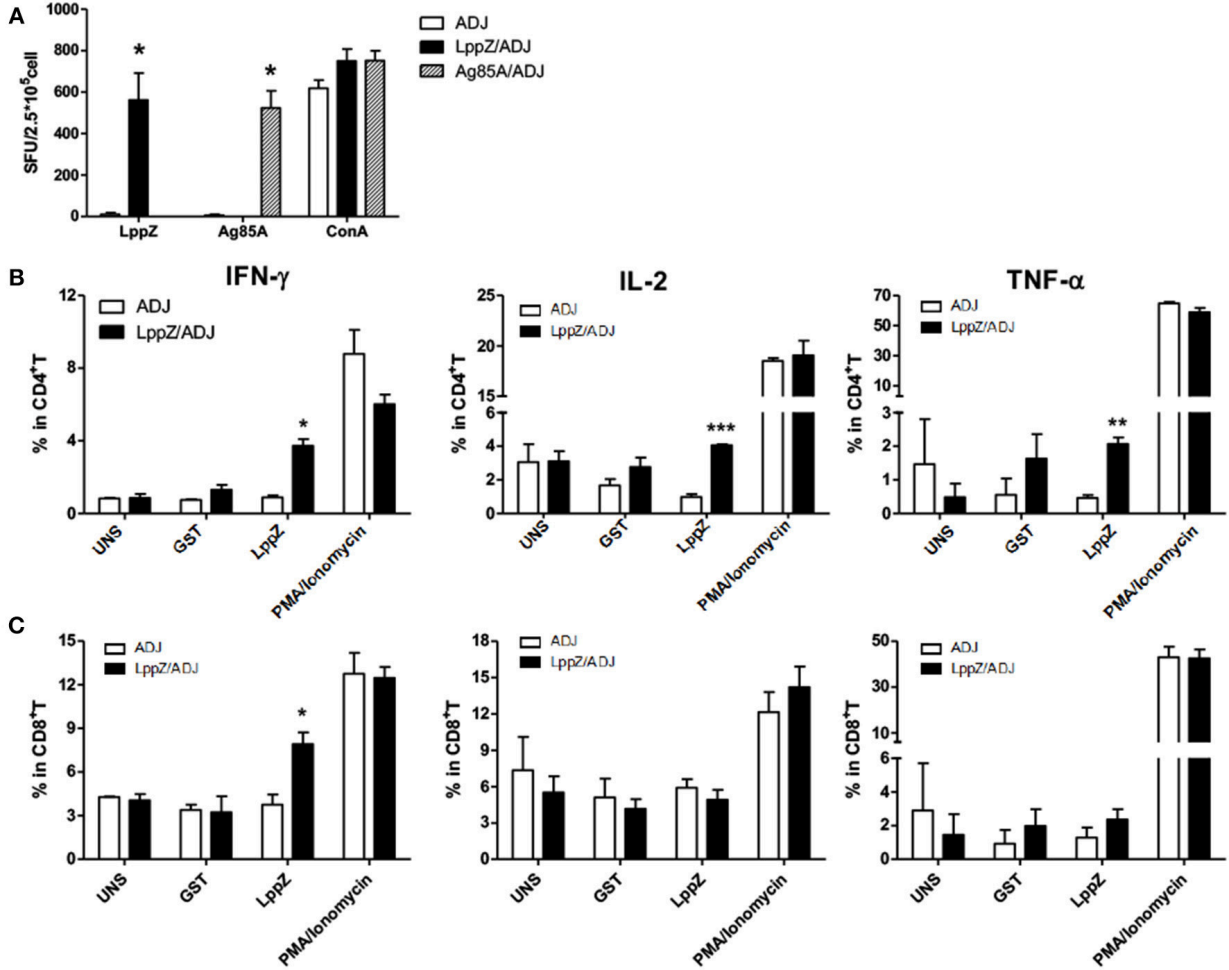
LppZ evokes increased IFN-γ and TNF-α release from splenocytes of immunized mice. **(A)** C57BL/6 female mice (6–8 weeks old) were immunized with LppZ (LppZ/ADJ) or Ag85A (Ag85A/ADJ) recombinant proteins emulsified with adjuvant, or adjuvant only (ADJ). Splenocytes from different immunization group were collected. For *ex vivo* stimulation, 2.5 × 10^5^ cells were incubated with LppZ (10 μg/mL) or Ag85A (10 μg/mL) for 20 h. Antigen-specific IFN-γ-producing cells were measured by ELISpot assay. ConA was treated as a positive control. **(B,C)** 5 × 10^5^ cells were incubated with LppZ (10 μg/mL) for 20 h. Antigen-specific IFN-γ, TNF-α, and IL-2 producing CD4^+^ **(B)** or CD8^+^ **(C)** T cells from the ADJ or LppZ/ADJ group were measured by flow cytometry. PMA/Ionomycin was a positive control and GST was an irrelevant control. UNS: without *ex vivo* antigen stimulation. *p < 0.05, **p < 0.01, ***p < 0.001.

*M.tb*-specific Th1 immune responses are considered to play critical roles in the protection against TB (26). The ability of LppZ to trigger efficient T cell responses was further investigated. C57BL/6 mice were immunized with LppZ and Ag85A was used as an experimental control (Supplementary Figure 5a). Subsequent analysis of antigen-specific cellular responses in the spleens showed that LppZ was able to induce the generation of antigen-specific IFN-γ producing cells (Figure 3A, black bar), to a degree similar to Ag85A immunization (Figure 3A, twill bar).

T cells from the spleens of LppZ immunized mice were further defined as functional subsets based on antigen-specific cytokine release. No significant differences were observed in the absolute numbers of CD4^+^ or CD8^+^ T cells with or without LppZ immunization (data not shown). However, LppZ could trigger antigen-specific CD4^+^ and CD8^+^ T cell responses after immunization: more LppZ-specific Th1-type CD4^+^ T cells that released cytokines IFN-γ, IL-2, and TNF-α were induced in LppZ-immunized mice when compared to those in the ADJ group (Figure 3B). LppZ evoked the generation of more IFN-γ releasing CD8^+^ T cells than ADJ did alone (Figure 3C). There were no significant differences between LppZ- and ADJ-injected groups in the frequencies of cytokine-releasing T cells upon stimulation with GST (an irrelevant control to exclude non-specific responses induced by trace contaminants in *E.coli*-derived proteins) or upon PMA/Ionomycin stimulation (positive control).

**Figure 4:**
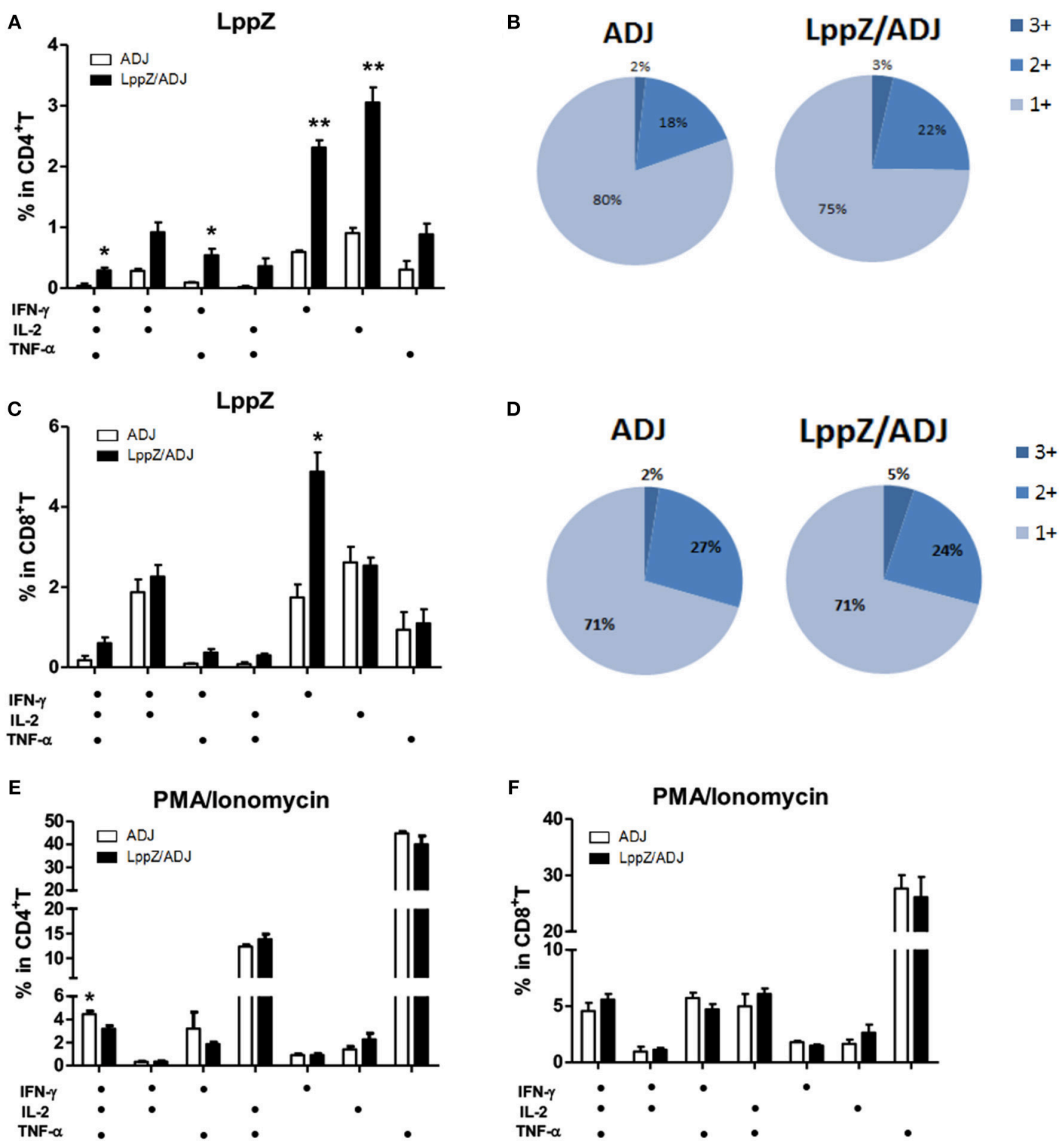
Polyfunctionality of CD4^+^ or CD8^+^ T cells responding to LppZ in immunized mice. Splenocytes from each mouse were collected and stimulated *ex vivo* with LppZ or PMA/Ionomycin. Frequencies of antigen-specific CD4^+^ **(A)** or CD8^+^ **(C)** T cells expressing single and multiple cytokines were analyzed by flow cytometry. Single or multiple cytokine production is shown on the x-axis of the bar chart (*n* = 3–6 mice per group). The percentages of CD4^+^ **(B)** and CD8^+^ **(D)** T cell expressing single (1+), double (2+) or triple (3+) cytokines are represented as pie charts. **(E,F)**: frequencies of cytokine-producing CD4^+^ **(E)** or CD8^+^ **(F)** T cells upon PMA/Ionomycin stimulation *p < 0.05, **p < 0.01.

Antigen-specific polyfunctional T cells are those releasing two or three cytokines simultaneously. They are considered to be the main fighters against *M.tb* infection (27, 28). LppZ-specific polyfunctional T cells were compared between LppZ-immunized and control mice. It was obvious that more IFN-γ^+^IL-2^+^TNF-α^+^ CD4^+^ T cells and IFN-γ^+^TNF-α^+^ CD4^+^ T cells were detectable in LppZ-immunized mice than in the ADJ group. In addition, CD4^+^ T cells producing either IFN-γ or IL-2 alone were increased more by the LppZ vaccination (Figure 4A). Similarly, IFN-γ^+^CD8^+^ T cells were induced more by LppZ treatment when compared to ADJ group (Figure 4C). However, in ADJ- and LppZ-treated mice, the percentages of single, double and triple cytokine-producing cells were similar both in CD4^+^ (Figure 4B) and CD8^+^ T cells (Figure 4D). In addition, equivalent levels of single or double cytokine releasing cells were induced upon PMA/Ionomycin stimulation in both CD4^+^ and CD8^+^ T cells in the two groups (Figures 4E,F).

### LppZ Immunization Protects Lungs From Injury With Less Bacilli Load

**Figure 5:**
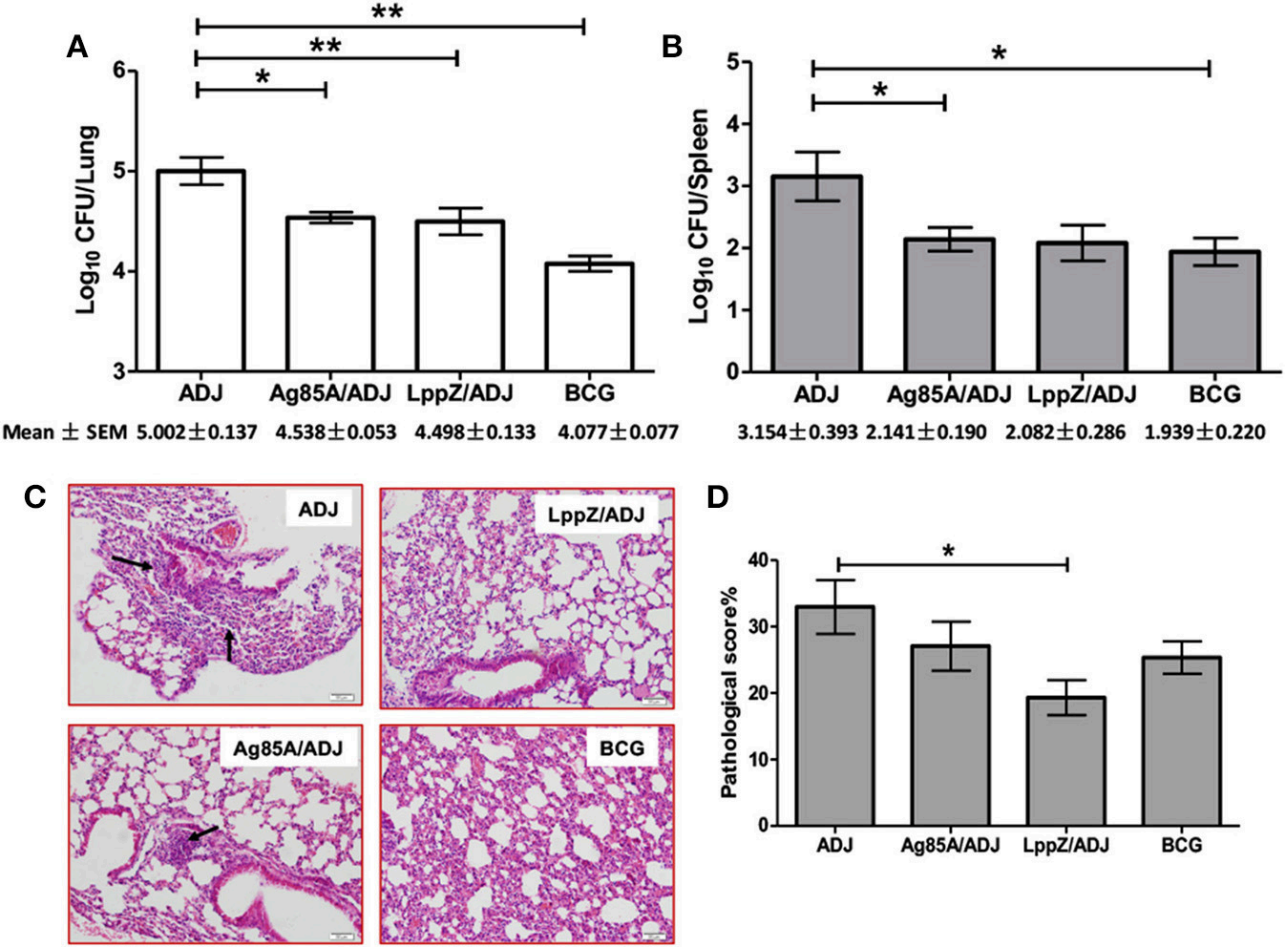
Bacteria load and lung histopathology after aerosol infection with virulent H37Rv strain. Six C57BL/6 female mice were immunized with purified Ag85A (Ag85A/ADJ) or LppZ (LppZ/ADJ) recombinant protein emulsified with adjuvant. Adjuvant only (ADJ) and BCG (BCG) immunization were used as controls. Four weeks after the last immunization, mice were challenged with the virulent *M.tb* strain H37Rv. Bacteria load in the lungs **(A)** and spleens **(B)** were enumerated and represented as the mean log_10_ CFU/organ ± SEM. Lung pathological scores **(C)** and representative pathological changes based on H&E staining (scale bar = 50 μm) **(D)** are indicated. *p < 0.05, **p < 0.01.

Four weeks after LppZ, Ag85A or BCG immunization, a low-dose aerosol inhalation challenge (100–200 CFUs per mice) with virulent *M.tb* H37Rv strain was used to determine the protective efficacy against infection (Supplementary Figure 5b). Four weeks after the challenge, the ADJ group presented the highest bacterial load in both lungs (Log_10_CFU = 5.002 ± 0.137) and spleens (Log_10_CFU = 3.154 ± 0.393), whereas BCG vaccination strongly reduced bacterial burdens developed in the two organs (Lung: Log_10_CFU = 4.077 ± 0.077; Spleen: Log_10_CFU = 1.939 ± 0.220). Notably, LppZ-immunized mice showed diminished bacterial loads in the lungs (Log_10_CFU = 4.498 ± 0.133) and spleens (Log_10_CFU = 2.082 ± 0.286) when compared to the ADJ group, an effect similar to that of Ag85A immunization (Lung: Log_10_CFU = 4.538 ± 0.053; Spleen: Log_10_CFU = 2.141 ± 0.290) and BCG vaccination (Figures 5A,B). Lung pathology of the challenged mice was also evaluated. Associated with the low bacilli loads in the lungs in LppZ-immunized mice, there was a less-pronounced lung inflammation characterized by less infiltration of immune cells in local regions and less alveolar tissue damage. In contrast, mice from the ADJ group showed much more severe inflammation (Figure 5C, arrow). LppZ-immunized mice exhibited the lowest pathological score in the lungs among the four groups based on H&E staining (Figure 5D). Our results thus demonstrated that LppZ immunization gave efficient protection against *M.tb* infection with less tissue damage.

### More LppZ-Specific Polyfunctional T Cell Responses Are Present in the Lungs After H37Rv Challenge in LppZ Immunized Mice

**Figure 6:**
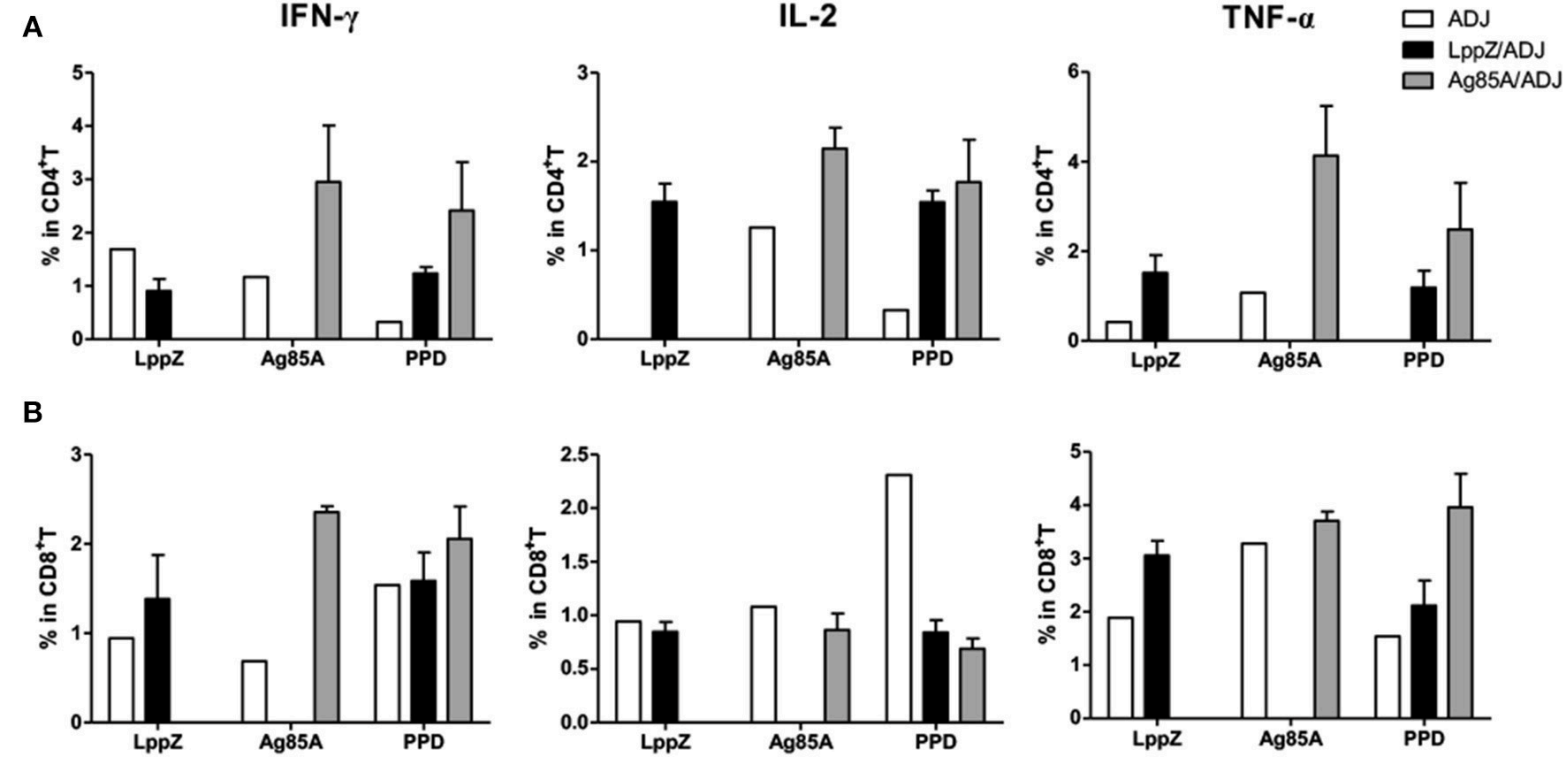
*M.tb* antigen-specific Th1-type cytokine production in CD4^+^ or CD8^+^ T cells from the lungs of virulent H37Rv-challenged mice. Four weeks after bacteria challenge, mice were sacrificed and lymphocytes from the lungs were collected. For *ex vivo* stimulation, 5 × 10^5^ cells from different groups (ADJ, LppZ/ADJ or Ag85A/ADJ) were incubated with 10 μg/mL LppZ, Ag85A or PPD respectively, for 20 h. Intracellular cytokine profile for IFN-γ, TNF-α and IL-2 in individual cells from different groups were measured by flow cytometry of CD4^+^ **(A)** or CD8^+^ **(B)** T cells.

Regional immune responses play important roles in the protection against *M.tb* infection (29). One lobe from the lungs of each immunized mouse after bacteria challenge was pooled and LppZ-specific T cell functionality was analyzed. Interestingly, LppZ induced different cytokine releasing patterns when compared to Ag85A. More LppZ-specific IL-2 and TNF-α producing CD4^+^ T cells were present in the lungs of the challenged LppZ-immunized mice when compared to the ADJ group, whereas Ag85A immunization led to more accumulation of Ag85A-specific IFN-γ^+^, IL-2^+^, and TNF-α^+^ CD4^+^ T cells in the lungs (Figure 6A). An increase in IFN-γ production in CD8^+^ T cells also was greater in both LppZ- and Ag85A-immunized mice than in the ADJ group (Figure 6B).

**Figure 7:**
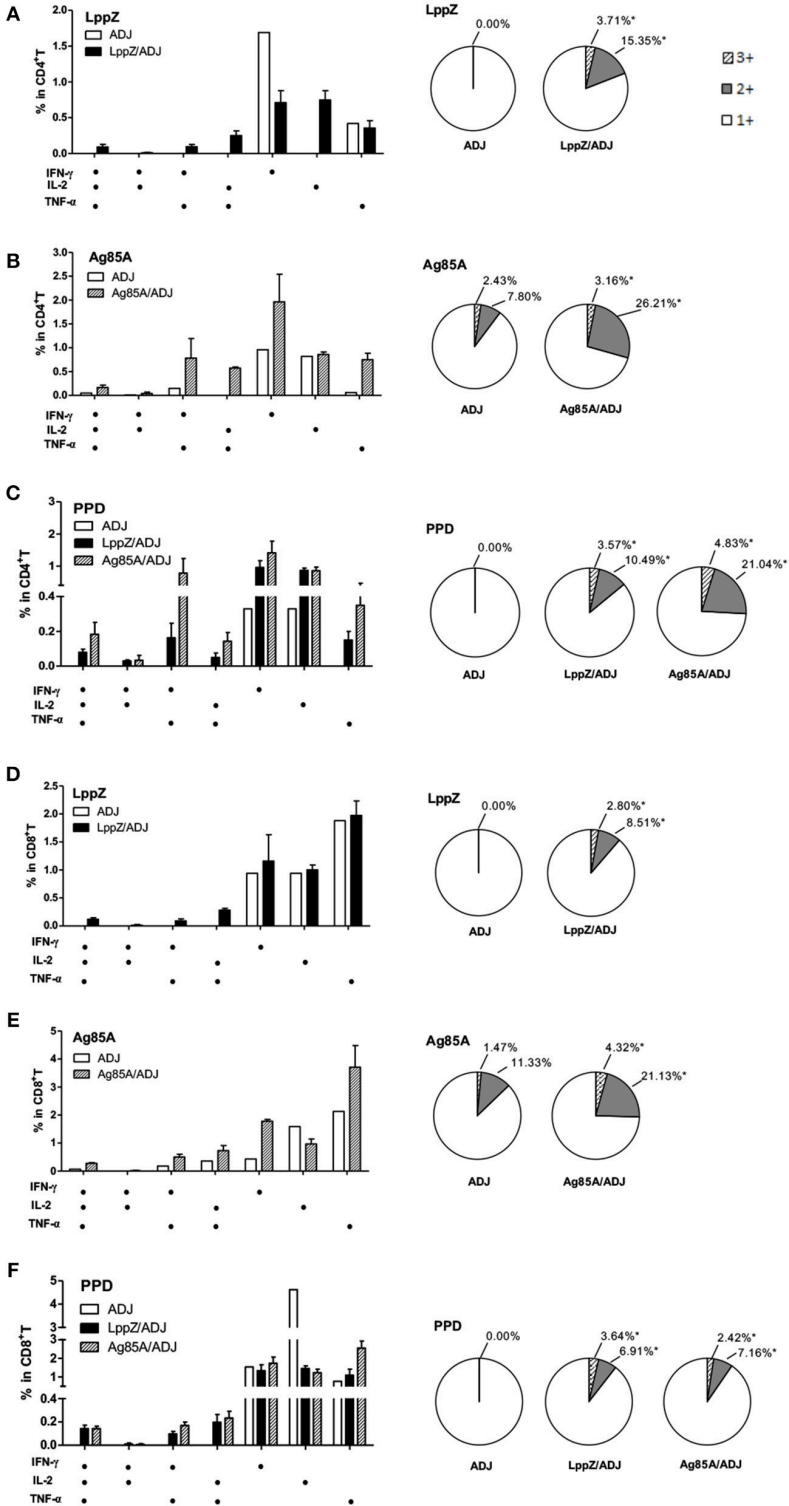
Polyfunctional CD4^+^ and CD8^+^ T cell distribution in the lungs of the immunized mice after H37Rv challenge. Pulmonary lymphocytes from different groups (ADJ, LppZ/ADJ or Ag85A/ADJ) were collected and stimulated *ex vivo* with LppZ **(A,D)**, Ag85A **(B,E)** or PPD **(C,F)**. Frequencies of antigen-specific CD4^+^ or CD8^+^ T cells producing single or multiple cytokines were analyzed by flow cytometry. Single or multiple cytokine production was shown on the x-axis of the bar chart. The percentages of T cells expressing single (1+), double (2+) or triple (3+) cytokines were represented as pie charts. *p < 0.05.

Antigen-specific polyfunctional T cells in the lungs of the H37Rv-challenged mice were also analyzed. LppZ immunization significantly increased the frequencies of multiple cytokine-releasing CD4^+^ and CD8^+^ T cells. In contrast, in challenged mice that had received ADJ alone, all LppZ-specific T cells produced single cytokines (Figures 7A,D). In challenged Ag85A-immunized mice, both Ag85A-specific double and triple cytokine releasing CD4^+^ and CD8^+^ T cells were more abundant in the lungs than in the ADJ control mice (Figures 7B,E). PPD-specific cellular immune responses, either CD4^+^ or CD8^+^ T cells, in the lungs after bacteria challenge were also compared between LppZ and Ag85A immunized mice. The frequencies of PPD-specific polyfunctional T cells in challenged LppZ- and Ag85A-immunized mice were higher than in ADJ control mice and were similar between the two groups (Figures 7C,F). These results revealed that LppZ immunization generated regional antigen-specific T cell immunity after bacterial challenge, which was similar to Ag85A immunization.

## Discussion

Because of the declining protection from BCG immunization in adult populations, the prime-boost strategy with a subunit vaccine is probably appropriate and calls for the identification of new candidate antigens (30–32). We propose LppZ as such a candidate. Owing to their cellular distribution and secreted nature, the LppZ lipoprotein family mediates bacterial interactions with the hosts through cell adhesion, invasion and transmembrane signaling (33). As investigated here, LppZ exhibited strong immunoreactivity in cellular responses in addition to the high humoral IgA responses in TB patients that were reported previously (21), confirming its dominant antigenicity among mycobacterial antigens.

Several lipoproteins previously have been previously reported to be involved in immunosuppressive processes. For instance, LpqH (19-kDa antigen), LprG and LprI exerted an immunosuppressive function at the innate immunity stage through toll-like receptors (34–39). This might help bacteria to escape from the initial immune surveillance. On the contrary, we have observed in this study that LppZ induced more dramatic innate immunity in the air pouch model with rapid recruitment of neutrophils at 4 h and the accumulation of macrophages and neutrophils in the pouches at 24 h. In the innate immune responses that follow *M.tb* infection, neutrophils and macrophages are the main components possessing strong phagocytosis properties and they occur earlier than T cell immunity. Rapid recruitment of neutrophils is the very first event in the fight against bacterial pathogens. They are responsible for the initiation of necrosis within the granuloma (40), and undergo killing processes and degranulation in response to *M.tb* infection. However, macrophages are considered to be the component to restrict bacilli survival and proliferation with more efficacy (41). What is more, alveolar macrophages, constituting the majority of cells collected from bronchoalveolar lavage, exert anti-inflammatory functions to control infection in the lung (21). Considering the membrane localization of LppZ in the bacteria, the presence of LppZ probably favors the elimination of bacteria through inducing a robust innate immunity once the bacteria encounter innate cells.

It is widely accepted that *M.tb*-specific T cell immunity is crucial for the protection against *M.tb* infection, especially polyfunctional T cells with the capacity for producing multiple cytokines (28, 42, 43). On the one hand, IFN-γ is one of the key cytokines produced by CD4^+^ T cells during *M.tb* infection. Subsequent activation of macrophages by IFN-γ is involved in the protection against TB (44–46). The anti-mycobacterial function of CD8^+^ T cells also depends on IFN-γ (47). TNF-α, on the other hand, can stimulate the production of reactive nitrogen intermediates (RNIs), restrict mycobacterial growth within macrophages and prevent necrosis of macrophages (48, 49). IL-2 tends to promote the proliferation and maturation of T cells and plays an important role in the control of *M.tb* infection as well (20, 31, 42, 43). In the present work, mice immunized with LppZ exhibited a strong capacity to generate antigen-specific Th1 immune responses, which was largely consistent with efficient protection upon *M.tb* challenge. More interestingly, LppZ immunization exerted protection against *M.tb* challenge similar to BCG or Ag85A immunization but with less pulmonary damage present in the lungs (Figure 5). Analyzing the immunological patterns, it was apparent that the frequencies of polyfunctional CD4^+^ and CD8^+^ T cells were moderate in LppZ-immunized mice after challenge when compared to BCG or Ag85A immunization (Figure 7). Similarly, the generation of mono-cytokine producing CD4^+^ and CD8^+^ T cells was also less pronounced (Figure 6) suggesting that while the induction of Th1 immunity by LppZ immunization protects against *M.tb* infection it mediates moderate inflammatory reactions. The protective efficacy together with the low pulmonary injury seen after the challenge of LppZ-immunized mice supports the further development of LppZ as one of the antigens in subunit booster vaccines in the future.

Cumulatively, we have reported in this study that LppZ showed strong immunogenicity during *M.tb* infection in humans as well as in mice. In mice, it was apparently able to induce innate and adaptive immunity and exerted efficient protection against *M.tb* infection similar to the current BCG vaccine. Considering the difficulty in substituting recombinant BCG in countries that are using BCG as primary vaccination and the limitations of the candidate antigens for booster vaccine development, further studies on LppZ-mediated protection in prime-boost animal models are warranted.

## Ethics Statement

This study was carried out in accordance with the recommendations of the Ethical Committee of Shanghai Jiao Tong University School of Medicine with written informed consent from all subjects. All subjects gave written informed consent in accordance with the Declaration of Helsinki. The protocol was approved by the Ethical Committee of Shanghai Jiao Tong University School of Medicine.

## Author Contributions

YW, XF, HS, and GZ: conceived the project. YC, XF, and YW: designed experiments. YC, JX, YLi, Y-jX, YLiu, SW, and PJ: performed experiments. Y-qX and SL: collected samples and clinical data. YC, JX, YLi, Y-jX, XF, and YW: analyzed data. YC, JX, Y-jX, and YW: wrote the manuscript. YC, JX, SL, XF, and YW: Obtained funding. GZ, HS, and SL are senior authors.

### Conflict of Interest Statement

The authors declare that the research was conducted in the absence of any commercial or financial relationships that could be construed as a potential conflict of interest.
