# Supplementary material for: Mycobacterial Lipoprotein Z Triggers Efficient Innate and Adaptive Immunity for Protection Against Mycobacterium tuberculosis Infection
Source: Front Immunol. 2019 Jan 16;9:3190. doi: 10.3389/fimmu.2018.03190 (PMC6343430; doi:10.3389/fimmu.2018.03190)
Supplement: Supplementary file 1 [file Data_Sheet_1.pdf]

Supplementary Material

Mycobacterial Lipoprotein Z Triggers Efficient Innate and Adaptive Immunity for Protection Against *Mycobacterium tuberculosis* Infection

Yingying Chen[1†], Jia-ni Xiao[1†], Yong Li[1†], Yang-jiong Xiao[1†], Yan-qing Xiong[2], Ying Liu[1], Shu-jun Wang[1], Ping Ji[1], Guo-ping Zhao[3], Hao Shen[1,4], Shui-hua Lu[2], Xiao-yong Fan[2,5*] and Ying Wang[1,3*]

*** Correspondence:** Prof. Ying Wang, Ph.D.: ywang@sibs.ac.cn; Prof. Xiao-yong Fan, Ph.D.: xyfan008@fudan.edu.cn

Supplementary Figures

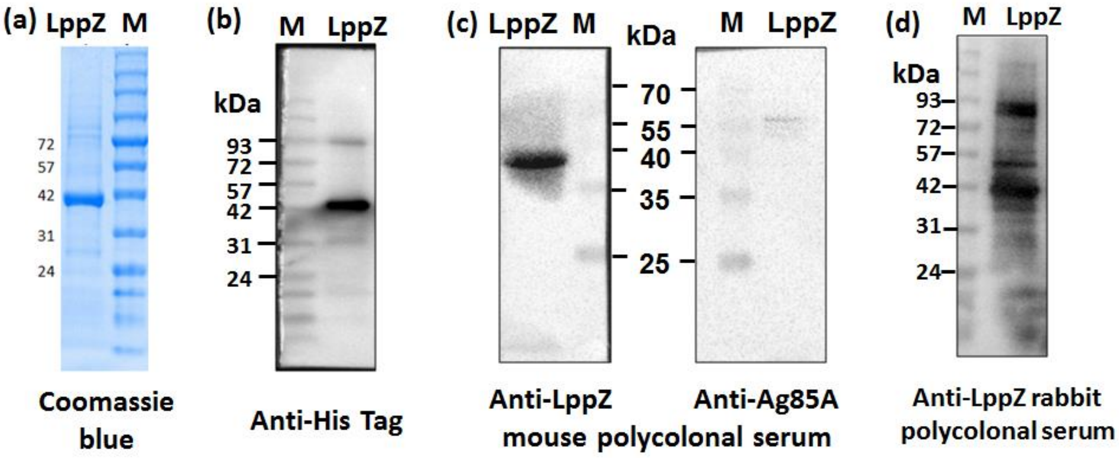

Supplementary Figure 1. Verification of *M.tb*-derived LppZ. (a) Coomassie blue staining of LppZ. Identification of purified LppZ recombinant protein by western blotting with anti-6His tag antibody (b), anti-LppZ (c, left panel) or anti-Ag85A (c, right panel) mouse polycolonal serum and anti-LppZ rabbit polycolonal serum (d). M: protein size marker.

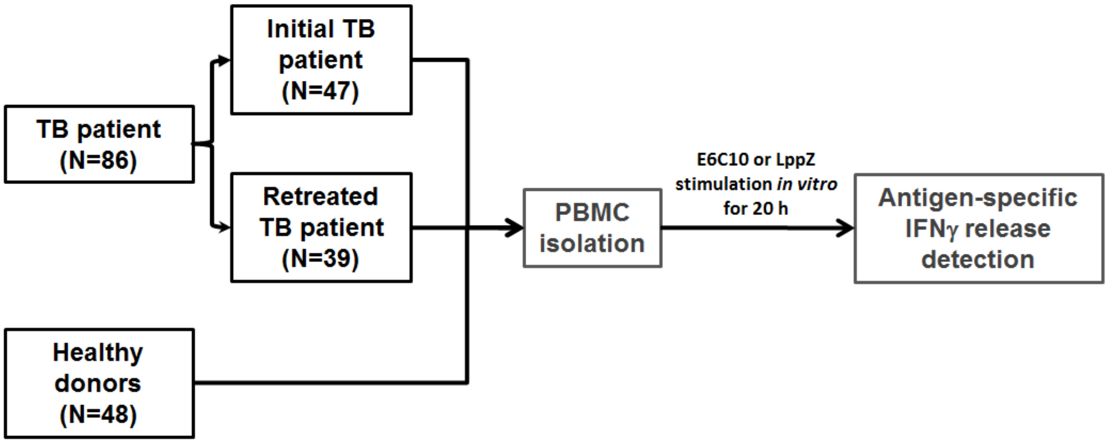

Supplementary Figure 2. Antigen-specific interferon-gamma (IFN-γ) release in human cohort. Human peripheral blood mononuclear cells (PBMCs) were isolated from TB patients or healthy donors by Ficoll-hypaque density-gradient centrifugation. Antigen-specific IFN-γ release was detected by enzyme-linked immunospot (ELISpot) assays. Freshly isolated cells were stimulated with 10 μg/mL purified LppZ or E6C10 fusion protein for 20 h. PHA served as positive control.

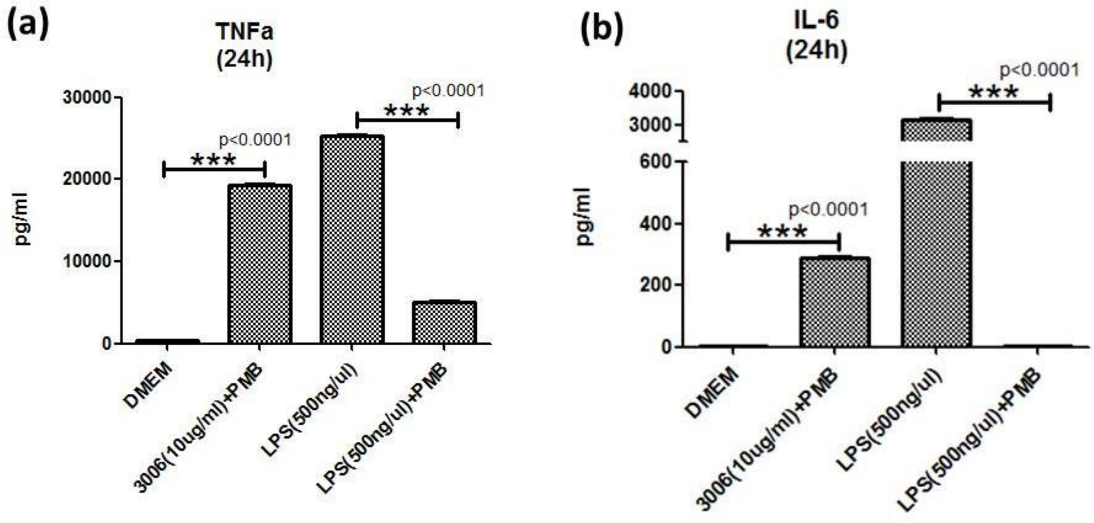

Supplementary Figure 3. Raw264.7 cells were incubated with DMEM medium, LppZ (10 μg/mL) plus Polymyxin B (PMB, 10 μg /mL), lipopolysaccharide (LPS, 500 ng/mL) or with LPS (500 ng/mL) plus PMB (10 μg /mL). After 24 h of incubation, cell culture supernatants were collected and TNF-α and IL-6 levels were measured by home-made ELISA. Concentrations of cytokines were represented as the mean ±SEM. ***: $p < 0.001$.

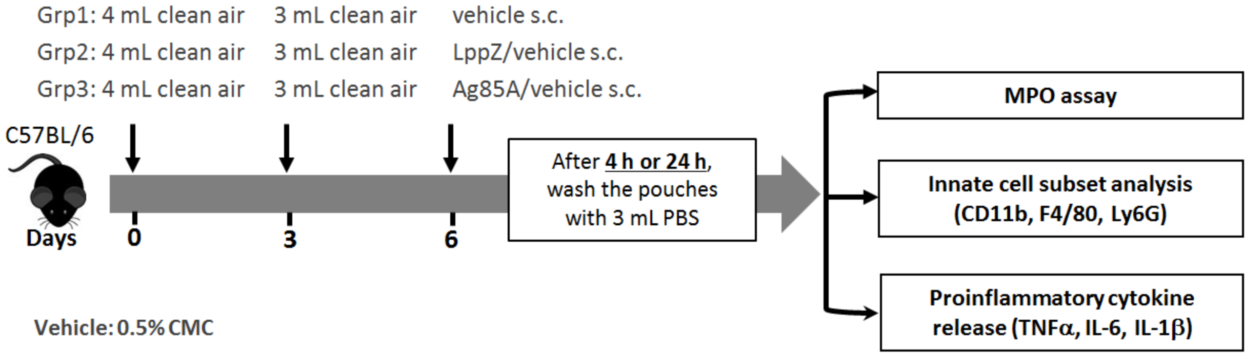

Supplementary Figure 4. Schema of dosal air pouch mouse model. 4 mL of sterile-filtered air was injected subcutaneously into the back of C57BL/6 female mice. After 3 days, the pouches were reinflated with 3 mL of sterile air. Three days later 1 mL 0.5% CMC mixed with 50 µg LppZ or Ag85A was injected. Injection of 1 mL 0.5% CMC served as a negative control. At 4 h or 24 h post-injection, mice were sacrificed and air pouches were lavaged with 3 mL sterile PBS. The lavage collected from the air pouches were utilized in MPO assay and detection of proinflammatory cytokine release. The inflitrated cell subsets were determined by flow cytometry.

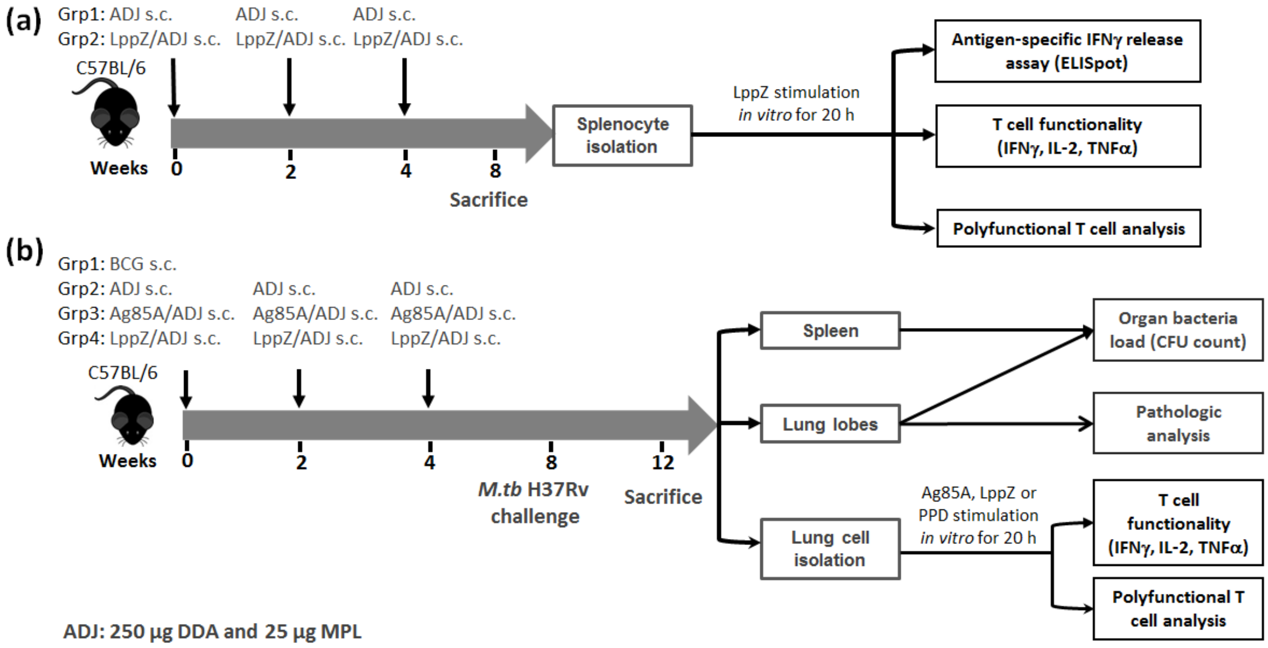

Supplementary Figure 5. Schema of antigen immunization and H37Rv challenge mouse model. (a) Female C57BL/6 mice were immunized subcutaneously with 10 μg LppZ emulsified with adjuvant mixture containing 250 μg dimethyl dioctadecylammonium bromide (DDA) and 25 μg monophosphoryl lipid A (MPL) in 200 μL PBS three times with 2-week intervals. Mice immunized with the adjuvant mixture only were served as the negative control. Four weeks after the final immunization, mice were subjected to immunological analysis, including antigen-specific IFNγ release and T cell functionality. (b) Female C57BL/6 mice were immunized subcutaneously with 10 μg LppZ or Ag85A emulsified with DDA/MPL adjuvant in 200 μL PBS three times with 2-week intervals. Mice immunized with one dose of 5×10^6 BCG Shanghai strain (Shanghai) served as a positive control. Four weeks later, all mice were exposed to 100-200 colony-forming unit (CFU) bacteria in an automatic inhalation system. Four weeks post-challenge, mice were sacrificed, and the spleens and part of the lobes of the lungs were subjected to enumerating the bacteria load. The rest lung lobes were subjected to hematoxylin and eosin (H&E) staining while immune cells from the lungs to immunological analysis (T cell functionality).
